# Immunogenicity of a Tripartite Cell Penetrating Peptide Containing a MUC1 Variable Number of Tandem Repeat (VNTR) and A T Helper Epitope

**DOI:** 10.3390/molecules23092233

**Published:** 2018-09-02

**Authors:** Nicole Brooks, Jennifer Hsu, Sandra Esparon, Dodie Pouniotis, Geoffrey A. Pietersz

**Affiliations:** 1 School of Medical Sciences, RMIT University, Plenty Road, Bundoora 3083, Victoria, Australia; backupburnet@gmail.com (N.B.); dodie.pouniotis@rmit.edu.au (D.P.); 2 Bio-Organic and Medicinal Chemistry Laboratory, Burnet Institute, 85 Commercial Rd, Melbourne 3004, Australia; j.hsu@sydney.edu.au (J.H.); sesparon@burnet.edu.au (S.E.); 3 Dendritic Cell Biology and Therapeutics Group, ANZAC Medical Research Institute, Institute of Haematology, Royal Prince Alfred Hospital, Missenden Rd, Camperdown, NSW 2050, Australia; 4 Department of Pathology, University of Melbourne, Parkville, Victoria 3010, Australia; 5 Department of Immunology, Monash University, Clayton, Victoria 3800, Australia; 6 Baker Heart and Diabetes Institute, Melbourne 3004, Australia; 7 College of Health and Biomedicine, Victoria University, Melbourne 3021, Australia

**Keywords:** penetratin, membrane translocating peptide, membrane penetrating peptide, vaccine, Mucin 1, immunogenicity, antigen delivery, immunotherapy, multiple antigen peptide, TLR agonist

## Abstract

Peptide-based vaccines for cancer have many advantages however, for optimization these immunogens should incorporate peptide epitopes that induce CD8, as well as CD4 responses, antibody and long term immunity. Cell penetrating peptides (CPP) with a capacity of cytosolic delivery have been used to deliver antigenic peptides and proteins to antigen presenting cells to induce cytotoxic T cell, helper T cell and humoral responses in mice. For this study, a tripartite CPP including a mucin 1 (MUC1) variable number of tandem repeat (VNTR) containing multiple T cell epitopes and tetanus toxoid universal T helper epitope peptide (tetCD4) was synthesised (AntpMAPMUC1tet) and immune responses investigated in mice. Mice vaccinated with AntpMAPMUC1tet + CpG show enhanced antigen-specific interferon-gamma (IFN-γ) and IL-4 T cell responses compared with AntpMAPMUC1tet vaccination alone and induced a Th1 response, characterised by a higher ratio of IgG2a antibody/IgG1 antibodies. Furthermore, vaccination generated long term MUC1-specific antibody and T cell responses and delayed growth of MUC1^+ve^ tumours in mice. This data demonstrates the efficient delivery of branched multiple antigen peptides incorporating CPP and that the addition of CpG augments immune responses.

## 1. Introduction

Despite encouraging results in some clinical trials, cancer vaccines are yet to have an impact on cancer [1,2]. Many strategies specific for a particular tumour associated antigen (TAA), including single or multiple peptides with or without ex vivo dendritic cells (DC) pulsing, DC targeting immunogens with or without adjuvants such as a toll-like receptor (TLR) agonist have been used [2,3,4,5,6]. To overcome some of the limitations of previous peptide vaccine strategies it is necessary to (1) incorporate both CD8 cytotoxic T cell (Tc) and CD4 T helper cell (Th) epitopes to promote the generation of long term immunity; (2) MHC restriction must be considered to allow application to the wider population and; (3) consideration to the most effective adjuvant to enhance immunogenicity [7,8,9,10].

Many TAA have been used as targets for cancer vaccines including Mucin 1 (MUC1), MAGE, NY-ESO-1, Her-2/neu and others [11,12]. MUC1, is a type I transmembrane glycoprotein with a protein core consisting of a 20 amino acid variable number of tandem repeat (VNTR) region (PGSTAPPAHGVTSAPDTRPA), which is frequently over-expressed by adenocarcinoma cells at levels 10–100 times greater than on normal tissues [13,14]. In a 2009 National Cancer Institute pilot project prioritizing 75 cancer antigens, MUC1 was ranked second as an immunotherapeutic cancer vaccine target antigen [15]. The 20 amino acid repeat of MUC1 contains T cell epitopes, that can be presented by many mouse H-2 haplotypes, including H-2K^b^, human HLA-A2.1, A1, A3, A11, a Th peptide that can be presented by HLA-DR3 and B cell epitopes. MUC1 peptide and protein based vaccines have been investigated in many clinical trials in numerous formulations [14,16,17,18].

Cell penetrating (CPP) or membrane translocating peptides (MTP) are a group of cationic peptides that have the ability to enter the cytoplasm of cells [19]. These peptides have been used to deliver a number of cargos such as proteins, peptides, DNA, RNA, drugs and virus particles into cells [19,20,21,22]. For these purposes 2 CPP, TAT from the human immunodeficiency virus transactivator of transcription protein and penetratin from the *Drosophila Antennapedia* domain have been used extensively. The *Drosophila Antennapedia* DNA binding domain contains 60 amino acids and consists of 3 α-helices, and the 16 amino acid peptide penetratin (RQIKIWFQNRRMKWKK; Antp) with internalizing activity is within the third helix.

The cell penetrating property of these peptides have been utilized to deliver antigenic peptide and proteins into any antigen presenting cell including DCs for the development of vaccine delivery systems [4,23,24,25,26,27,28,29,30,31,32,33,34,35,36,37,38,39]. For this purpose, CPP peptides chemically conjugated to protein antigens or linear synthetic peptides of CPP fused in tandem with cytotoxic (Tc) or helper (Th) T cell epitopes have been used. Mice immunised with these constructs generated antigen-specific CD4, CD8 or mixed responses and were protected from a subsequent tumor challenge [4,30,40]. To increase the versatility of penetratin-based immunogens, it has been chemically linked a 4-arm multiple antigen peptide (MAP) with 4 ovalbumin (OVA) H2-K^b^ Tc epitope peptides (SIINFEKL) to the CPP, which resulted OVA-specific immunity and protection from tumour challenge in mice [24]. In the current study, the immunogenicity of a novel tripartite peptide incorporating penetratin, tetanus toxoid universal CD4 epitope peptide and a single VNTR of the MUC1 antigen is investigated.

Toll-like receptors (TLR) are a family of conserved pattern recognition receptors that recognizes specific microbial patterns. To enhance immunogenicity, simultaneous delivery of an adjuvant along with tumour antigens represents an effective approach to vaccination [41]. Unmethylated CpG DNA is recognized by TLR9, poly(I:C) recognised by TLR3, imidazoquinolines imiquimod and resiquimod recognizing TLR7/8, TLR4 and TLR2 have been used extensively. A few studies have investigated the potential of CPP-based immunogens to enhance immunogenicity [24,37,42].

In the current study, we demonstrate that a tripartite branched CPP incorporating the H-2K^b^ (SAPDTRPAP)- and HLA-A2 (STAPPAHGV)-restricted CTL epitopes from the MUC1 tumour antigen with the universal Th epitope tetanus toxoid (tetCD4) is internalised into DC in vitro, as well as in vivo. The tripartite peptide when combined with CpG induced T cell responses as measured by IFNγ and IL4 ELISpot analysis, in vivo CTL and protected mice from a tumour challenge. Additionally, long term MUC1-specific antibody and T cell responses were generated by the tripartite peptide.

## 2. Results

### 2.1. Biochemical and Immunochemical Characterisation of the Tripartite Peptide Containing Penetratin, MUC1 VNTR and Tetanus Toxin CD4 Peptide

**Figure 1:**
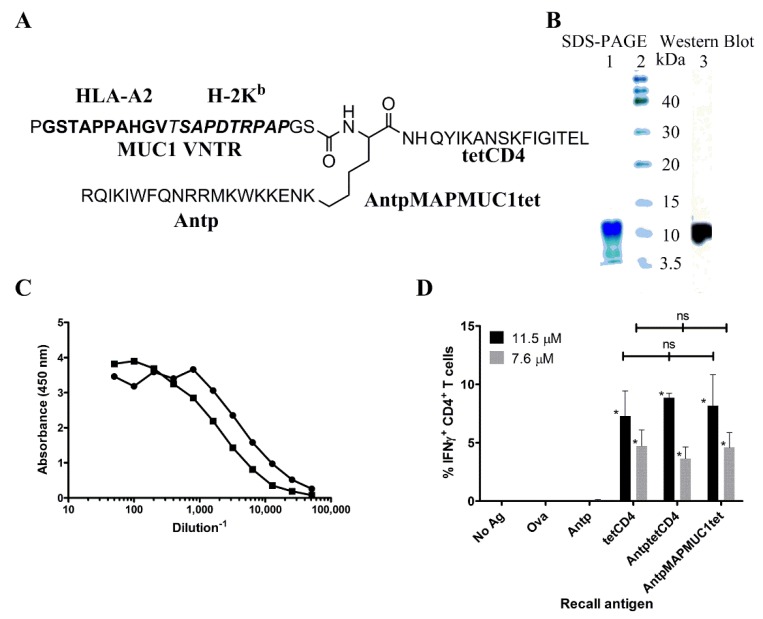
(**A**) Structure of the AntpMAPMUC1tet immunogen. The HLA-A2 restricted CTL epitope and the H2-K^b^ epitope of the mucin 1 (MUC1) variable number of tandem repeat (VNTR) is denoted in bold type. (**B**) SDS-PAGE and western blot analysis of AntpMAPMUC1tet (lane 1), molecular weight markers (lane 2). Anti-MUC1 antibody, 1.3.14 was used for western blot analysis (lane 3). (**C**) Binding of anti-MUC1 antibodies to AntpMAPMUC1. AntpMAPMUC1tet was coated onto a 96-well microtitre plate and bound peptide detected with anti-MUC1 antibody, BC2 recognizing the DTR epitope (●) and 1.3.14 antibody recognizing the APPAH epitope (■) in the tripartite peptide, AntpMAPMUC1tet. (**D**)Tetanus toxoid CD4 T cell epitope (tetCD4) in AntpMAPMUC1tet is processed and presented by human MoDC to tetCD4-specific human T cell lines. MoDC were pulsed with equimolar concentrations (7.6 or 11.5 uM) of tetCD4, AntptetCD4, AntpMAPMUC1tet or Antp, OVA (non-specific control antigens) for 14 hr before the addition of responder cells for 15 h. Golgistop was added for a further 4 h before the cells were stained for CD4 and intracellular IFNγ. MoDC alone with or without OVA or Antp were used as negative controls for non-specific IFNγ production. Values represent IFNγ production in tetCD4-specific T cells ± SEM stimulated by pulsed MoDC from 3 different donors. * *p* < 0.05.

The tripartite peptide consisting of penetratin (RQIKIWFQNRRMKWKKENK), tetanus toxoid universal T cell epitope (QYIKANSKFIGITEL) and a single VNTR from MUC1 (PGSTAPPAHGVTSAPDTRPAPGS) (Figure 1A) was synthesised by solid phase peptide synthesis and had a purity of >85% and expected mass of 6846.77 by mass spectroscopy.

Analysis of the tripartite peptide by SDS-PAGE electrophoresis indicated that it migrated at an apparent molecular weight of ~10 kDa (Figure 1B). This discrepancy is probably due to the absence of a tertiary structure and not migrating at a desired molecular weight position determined by the globular protein molecular weight standards. Western blot analysis of the SDS-PAGE gel with the antibody 1.3.14 reactive with the APPAH sequence identified the band at ~10 kDa (Figure 1B). The antibodies 1.3.14 and BC2 with specificity to APPAH and DTR also bound the tripartite peptide in an ELISA confirming presence of MUC1K^b^ (SAPDTRPAP) and MUC1A2 (STAPPAHGV) peptide epitopes in AntpMAPMUC1tet (Figure 1C). The presence of the tetanus toxoid epitope was determined using a tetanus peptide-specific CD4 T cell line generated by in vitro priming of PBMC with the tetCD4 peptide. These bulk cultures contained ~40% tetCD4-specific T cells when stimulated with 20 µM tetCD4 (not shown). Autologous MoDCs pulsed with AntpMUC1tet stimulated the tetCD4-specific T cell line as detected by intracellular IFNγ secretion (Figure 1D) by flow cytometry. The levels of intracellular IFNγ secreted were not significantly different to that induced when tetCD4 peptide alone or when an AntptetCD4 fusion peptide was used for stimulation. No significant IFNγ was induced in tetCD4-specific T cells when stimulated by the Antp peptide, unpulsed cells or an irrelevant antigen, ovalbumin (OVA). These biochemical and immunochemical assays further confirmed the identity of the tripartite peptide for further immunological studies.

### 2.2. Internalisation of AntpMAPMUC1tet by Bone Marrow Derived Dendritic Cells (BMDC)

**Figure 2:**
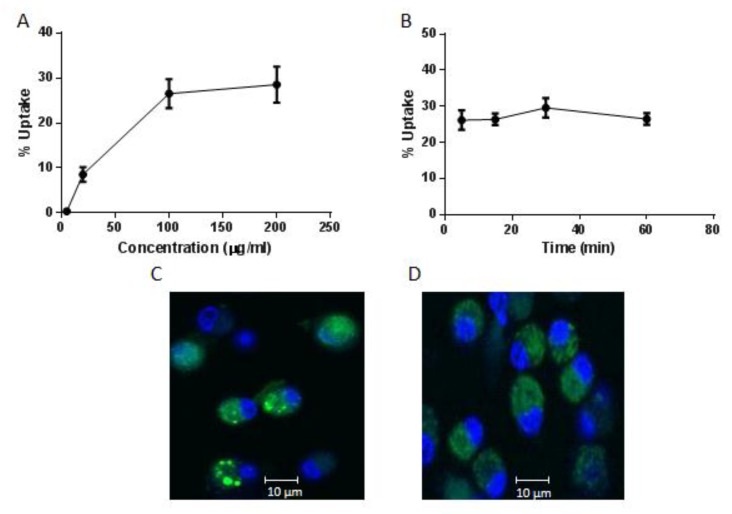
Uptake of AntpMAPMUC1tet peptide by dendritic cells (DC) in vitro. (**A**) DC were pulsed with AntpMAPMUC1tet peptide at either varying concentrations (5 to 200 µg/mL) for 60 min or (**B**) a constant dose of 100 µg/mL for set times between 5 and 60 min. Uptake was determined by flow cytometry as the percent surface staining subtracted from the percent intracellular staining (mean ± SEM, for 3 replicates). (**C**) DCs were incubated with 100 µg/mL Alexa-labelled AntpMAPMUC1tet (green) in chamber slides for 2 h, counterstained with Hoechst nuclear stain (blue), washed and visualized by confocal microscopy or (**D**) DC were pre-treated with 10 µg/mL cytochalasin D before AntpMAPMUC1tet pulsing.

The mechanism of cellular uptake of CPP has been an area of great interest. Cell fixation leads to the artefactual uptake of these peptides. The DTR sequence within the H2-K^b^ restricted peptide epitope is recognised by the BC2 antibody enabling detection of internalised AntpMAPMUC1tet peptide without chemical modification [23]. Thus, to assess the uptake of AntpMUC1tet by DC, surface and intracellularly localised peptide was measured by flow cytometry staining with the BC2 antibody. To investigate the internalisation of AntpMAPMUC1tet, DC were pulsed with varying concentrations of AntpMAPMUC1tet for 60 min (Figure 2A).

Moderate uptake of AntpMAPMUC1tet is seen at 20 µg/mL, with maximum uptake at 100 µg/mL and plateau at 200 µg/mL. To investigate kinetics of uptake DC were incubated at various times with AntpMAPMUC1tet (5–60 min) at a fixed concentration (100 µg/mL) and internalisation determined. Rapid uptake was observed within 5 min, remaining constant for 60 min (Figure 2B).

To further investigate the intracellular uptake by BMDC, Alexa-488-labelled AntpMAPMUC1tet was used for confocal microscopy [43]. AntpMAPMUC1tet containing vesicles were detectable after 2 h incubation with BMDC (Figure 2C,D). Addition of cytochalasin D which disrupts actin microfilaments substantially inhibited uptake leading to diffuse fluorescence indicating the role of macropinocytosis or phagocytosis in AntpMAPMUC1tet peptide internalisation by BMDC (Figure 2D). Therefore, the branched CPP containing immunogen is rapidly internalised into DC similar to the linear CPP or covalently linked immunogens [23,43,44].

### 2.3. AntpMAPMUC1tet Targets Antigen Presenting Cells (APC) In Vivo

**Figure 3:**
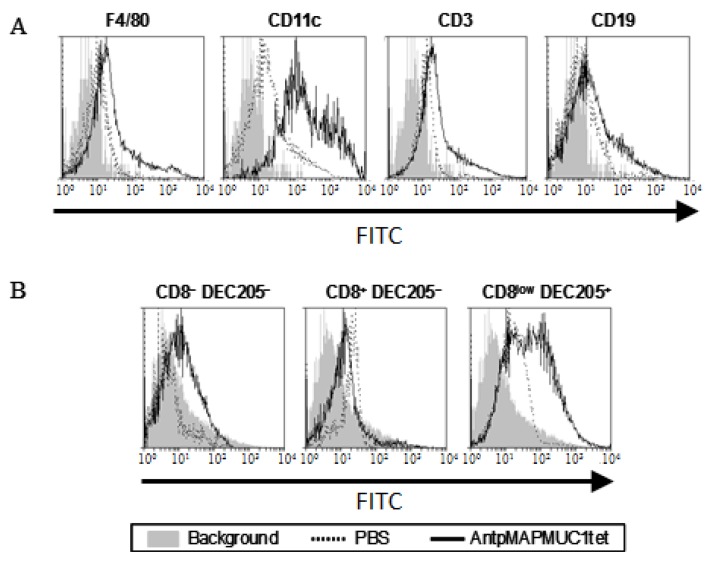
AntpMAPMUC1tet targets APC in vivo. Popliteal lymph nodes were isolated from mice (*n* = 4) 18 h after i.d. immunisation with PBS or FITC-labelled AntpMAPMUC1tet. (**A**) Lymph node cells were stained with anti-CD3, CD19, CD11c and F4/80 antibodies. (**B**) Cells were labelled with CD11c, CD8 and DEC205 to separate DC subsets and analysed by flow cytometry. Each labelled population was analysed for FITC fluorescence intensity. Results are depicted as histograms representative of two separate experiments.

To examine the in vivo localisation of AntpMAPMUC1tet, lymph node cells isolated from mice 18 h after FITC-conjugated AntpMAPMUC1tet injection were analysed for FITC fluorescence intensity by flow cytometry [45]. As shown in Figure 3A F4/80^hi^ macrophages, CD3^+^ T cells, and CD19^+^ B cells show some FITC-AntpMAPMUC1tet binding. However, CD11c^hi^ DC show considerably higher uptake suggesting that AntpMAPMUC1tet preferentially targets professional APC after i.d. immunisation. Based on CD8 and DEC205 expression, CD11c^hi^ DCs can be further divided into three subpopulations: CD8^−^ DEC205^−^ (immature), CD8^+^ DEC205^−^ (immature) and CD8^low^ DEC205^+^ DCs (mature) [46]. To further investigate the uptake by DC, all three subsets were analysed by flow cytometry indicating that AntpMAUC1tet target the mature CD8^low^ DEC205^+^ DCs subset (Figure 3B). Thus, AntpMAPMUC1 is internalised into DC in vitro (above), as well as in vivo.

### 2.4. Addition of CpG to AntpMAPMUC1tet Induces Maturation of DCs In Vivo

**Figure 4:**
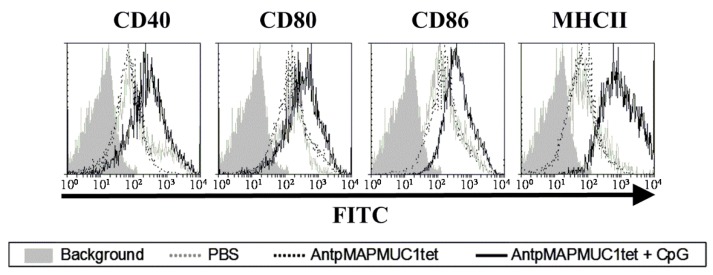
DC maturation in vivo by AntpMAPMUC1tet with and without CpG. C57BL/6 mice were injected i.d. with PBS, AntpMAPMUC1tet or AntpMAPMUC1tet + CpG. 18 h later popliteal lymph nodes were isolated and stained with the DC marker CD11c and maturation markers (CD40, CD80, CD86, and MHC class II) and assessed by flow cytometry. Representative histogram plots are shown (*n* = 3).

The activation of TLR on DCs by their corresponding ligands results in the induction of a mature phenotype of DCs, inducing the up regulation of MHC and co-stimulatory (CD80 and CD86) molecules, thus potentially augmenting T cell stimulatory capacity [47,48]. To assess the maturation of DC in vivo C57BL/6 mice were injected i.d. with AntpMUC1tet with or without CpG on the footpads and 18 h later popliteal lymph nodes were removed, and cells stained with the DC marker CD11c and maturation markers (CD40, CD80, CD86, and MHC class II) assessed by flow cytometry. Analysis indicated that i.d. injection of AntpMAPMUC1tet alone does not mature DC in vivo, only AntpMAPMUC1tet + CpG immunisation matures DCs in vivo, characterised by increased expression of CD40, CD80, CD86, and MHC class II (Figure 4).

Consistent with other CPP immunogens AntMAPMUC1tet do not mature DC however, addition of CpG activated DC resulting in upregulation of costimulatory molecules in vivo.

### 2.5. AntpMAPMUC1tet Induces MUC1-Specific T Cell Responses In Vivo in C57BL/6 Mice

To determine whether AntpMAPMUC1tet was able to induce T cell responses in vivo, mice were immunised on days 0, 10 and 17 with PBS, 100 µg AntpMAPMUC1tet with or without 50 µg CpG. Antigen specific IFN-γ and IL-4 secretion by splenocytes from mice were determined by ELISpot analysis 14 days after last immunisation. 

**Figure 5:**
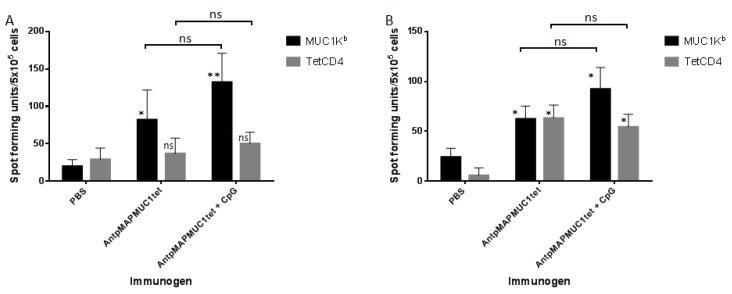
In vivo IFN-γ and IL4 response to AntpMAPMUC1tet and AntpMAPMUC1tet + CpG in C57BL/6. C57BL/6 mice were injected i.d. on day 0, 10 and 17 with PBS, 100 µg AntpMAPMUC1tet and AntpMAPMUC1tet + CpG. Number of IFN-γ secreting cells (**A**) and IL4-secreting cells (**B**) to MUC1K^b^ (SAPDTRPAP) and tetCD4 antigens were analysed by ELISpot assay. ConA (1 µg/mL) was used as an internal positive control (not shown). Results are shown as mean spot-forming units (SFU)/5 × 10^5^ cells ± SEM. Results are representative of three separate experiments. * *p* < 0.05, ** *p* < 0.001.

Mice immunised with AntpMAPMUC1tet generated significant responses (*p* < 0.05) to MUC1K^b^ (SAPDTRPAP) compared to PBS immunised group. Significant responses (*p* < 0.001) were also detected against SAPDTRPAP in the AntpMUC1tet + CpG group compared to PBS group however, the level of IFNγ was not significantly different to that in AntpMUC1tet alone group. No IFN-γ response was detected in any of the groups to the tetanus toxoid tetCD4 epitope (Figure 5A).

Analysis of IL-4 secretion by splenocytes revealed that both AntpMAPMUC1tet and AntpMAPMUC1tet + CpG immunisation generates significantly higher responses against tetanus toxoid T helper peptide (tetCD4) when compared to PBS control group (*p* < 0.05). Similarly, mice immunised with AntpMAPMUC1tet generated significantly higher responses (*p* < 0.05) against SAPDTRPAP (MUC1K^b^) compared to PBS group (Figure 5B). However, the IL4 response in the CpG groups were not significantly different to AntpMUC1tet alone.

### 2.6. AntpMAPMUC1tet Immunisation Induces Specific T Cell Responses In Vivo in HLA-A2 Transgenic Mice

**Figure 6:**
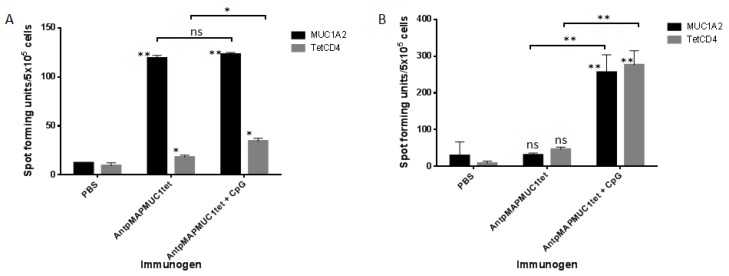
In vivo IFN-γ and IL4 response to AntpMAPMUC1tet and AntpMAPMUC1tet + CpG in HLA-A2 transgenic mice. HLA-A2 mice were injected i.d. on day 0, 10 and 17 with PBS, 100 µg AntpMAPMUC1tet and AntpMAPMUC1tet + CpG. Number of IFN-γ secreting cells (**A**) and IL4-secreting cells (**B**) to MUC1A2 (STAPPAHGV) and tetCD4 recall antigens were analysed by ELISpot assay. ConA (1 µg/mL) was used as an internal positive control (not shown). Results are shown as mean spot-forming units (SFU)/5 × 10^5^ cells ± SEM. Results are representative of three separate experiments. * *p* < 0.05, ** *p* < 0.001.

To establish if immunisation with AntpMAPMUC1tet could generate MUC1 HLA-A2.1-restricted STAPPAHGV-specific responses human MHC I, HLA-A2*0201/K^b^ mice were used. HLA-A2 transgenic mice were immunised i.d. with PBS, 100 µg AntpMAPMUC1tet or AntpMAPMUC1tet + CpG on days 0, 10 and 17 and IFN-γ and IL-4 responses measured 14 days after final immunisation. Pulsing splenocytes with the HLA-A2 STAPPAHGV peptide induced significant IFN-γ responses in both AntpMAPMUC1tet and AntpMAPMUC1tet + CpG groups (*p* < 0.001). Low but significant IFN-γ responses were also seen in groups against tetanus toxoid (Figure 6A). Analysis of IL-4 secretion by splenocytes from mice revealed only mice immunised with AntpMAPMUC1tet + CpG generated significant IL-4 responses to STAPPAHGV and tetanus toxoid (*p* < 0.001). No IL-4 response was detected in AntpMAPMUC1tet group compared to PBS (Figure 6B).

### 2.7. Combination of CpG with AntpMUC1tet Induces Enhanced In Vivo Antigen Specific Cytotoxic Killing

**Figure 7:**
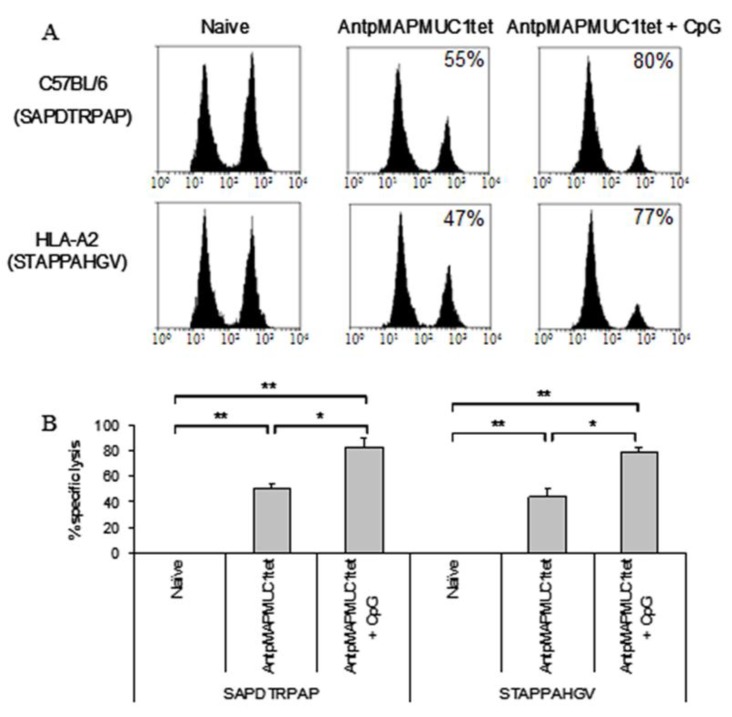
Cellular immune responses in AntpMAPMUC1tet immunised mice measured as in vivo CTL killing assays. (**A**) C57BL/6 or HLA-A2 mice were immunised i.d. with PBS, 100 µg AntpMAPMUC1tet or 100 µg AntpMAPMUC1tet + 50 µg CpG and the percent MUC1K^b^ or MUC1A2-specific lysis was determined eight days after immunisation calculated as: {[1 − (ratio CFSE^low^/CFSE^high^ of PBS mice/ratio CFSE^low^/CFSE^high^ of immunised mice)] × 100}. Representative histograms are shown. (**B**) Data is presented as mean % of killing ± SEM (*n* = 6). * *p* < 0.05, ** *p* < 0.001.

The capacity of both C57BL/6 and HLA-A2 transgenic mice to generate antigen specific killing in vivo following immunisation was assessed using in vivo CTL assays [24,44]. To detect MUC1K^b^ restricted lysis, groups of 6 C57BL/6 mice were injected with PBS, 100 ug AntpMUC1tet with or without CpG. Splenocytes from naïve mice were prepared, pulsed with SAPDTRPAP peptide and labelled with carboxyfluorescein diacetate succinimidyl ester (CFSE) at high or low concentrations and subsequently adoptively transferred into immunised mice. Mice pre-immunised with AntpMAPMUC1tet + CpG generated significantly greater lysis (55 ± 3% vs. 80 ± %, *p* < 0.05) than mice immunised with AntpMAPMC1tet alone (Figure 7A,B).

Likewise HLA-A2 transgenic mice immunised with AntpMAPMUC1tet + CpG showed significantly greater MUC1A2 STAPPAHGV in vivo lysis (47 ± 6% vs. 77 ± 3%, *p* < 0.05) than mice immunised with AntpMAPMUC1 (Figure 7A,B). Therefore, in C57BL/6 and HLA-A2 transgenic mice AntpMAPMUC1tet induces MUC1-specific T cells that secrete IFNγ when stimulated with H2-K^b^ and HLA-A2-restricted CTL epitopes (above) and furthermore the induced T cells are cytolytic in vivo.

### 2.8. AntpMAPMUC1tet + CpG Immunisation Delayed Tumour Growth in Prophylactic Tumour Challenge

**Figure 8:**
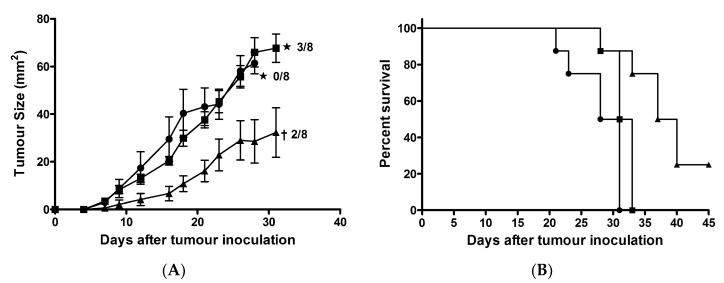
Tumour growth is delayed by immunization (**A**). C57BL/6 mice were immunised on days 0, 10 and 17 with PBS (●), 100 µg AntpMAPMUC1tet (■) or 100 µg AntpMAPMUC1tet + 50 µg CpG (▲) then inoculated subcutaneously 7 days after final immunisation with 2 × 10^5^ B16-MUC1 melanoma cells into the abdomen. Tumour growth was recorded. Data showing the product of individual perpendicular measurements (mm^2^) and days post tumour inoculation. Number of tumour-free mice (†) and number of surviving mice (★) is also shown. Kaplan–Meier survival curves for each immunised group are shown (**B**).

To evaluate the efficacy of AntMAPMUC1tet in tumour protection, groups of C57BL/6 mice (*n* = 8) were immunised with PBS, 100 µg AntpMAPMUC1tet or 100 µg AntpMAPMUC1tet + 50 µg CpG on days 0, 10 and 17. Seven days, after the last immunisation, mice were challenged subcutaneously with 2 × 10^5^ B16-MUC1 cells on the abdomen [44,49]. Immunisation of mice with AntpMAPMUC1tet did not retard B16-MUC1 tumour growth. In contrast, mice immunised with AntpMAPMUC1tet + CpG exhibited significantly delayed tumour growth (Figure 8).

On day 31, 2 out of 8 mice in AntpMAPMUC1tet + CpG immunised group were tumour free, whilst all control PBS injected mice and 5 of 8 AntpMAPMUC1tet immunised mice succumbed to tumour and had to be culled. The mean tumour size on day 28 in AntpMAPMUC1tet + CpG group was significantly smaller (*p* < 0.05) at 28.6 ± 9.1 mm^2^, whilst tumour size in control and AntpMAPMUC1 alone group was 61.5 ± 4.5 and 66.0 ± 6.1 mm^2^ respectively. In addition, mice immunised with AntpMAPMUC1tet survived significantly longer (38.5 days) (*p* = 0.006) than controls (29.5 days) and AntpMAPMUC1tet (32 days) immunised mice (Figure 8B).

### 2.9. Induction of Long Term Immunity is Enhanced by CpG

The induction of long term antigen specific T cell responses in vivo following immunisation C57BL/6 mice was assessed by ELISpot and in vivo CTL assays performed 40 days after the last immunization. Mice were injected on days 0, 10, and 17 and i.d. with PBS, 100 µg AntpMAPMUC1tet or 100 µg AntpMAPMUC1tet with 50 µg CpG and T cell responses, measured 40 days after final immunisation.

**Figure 9:**
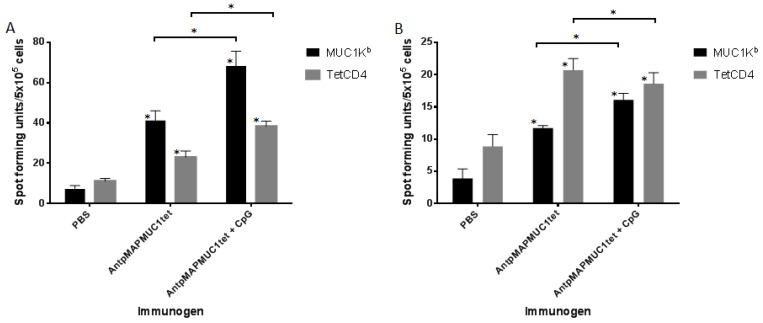
Long term in vivo IFN-γ and IL-4 responses to AntpMAPMUC1tet and AntpMAPMUC1tet + CpG. C57BL/6 mice were injected i.d on day 0, 10 and 17 with PBS, 100 µg AntpMAPMUC1tet and AntpMAPMUC1tet + CpG. Number of IFN-γ secreting cells (**A**) and IL4-secreting cells (**B**) to MUC1K^b^ (SAPDTRPAP) and tetCD4 antigens were analysed by ELISpot assay. ConA (1 µg/mL) was used as an internal positive control (not shown). Results are shown as mean spot-forming units (SFU)/5 × 10^5^ cells ± SEM. Results are representative of three separate experiments. * *p* < 0.05.

Mice immunised with AntpMAPMUC1tet alone revealed IFN-γ responses against SAPDTRPAP and tetCD4 compared to control mice. Immunisation with AntpMAPMUC1tet + CpG induced significantly higher levels of IFN-γ responses against SAPDTRPAP (MUC1K^b^) and tetCD4 compared to AntpMUC1tet mice (*p* < 0.05) (Figure 9A). AntpMAPMUC1tet and AntpMAPMUC1tet + CpG induced significant IL-4 responses to SAPDTRPAP and tetCD4 (Figure 9B). It was also apparent that the addition of CpG enhanced IFNγresponses to MUC1K^b^ and tetCD4 and IL4 responses to MUC1K^b^.

**Figure 10:**
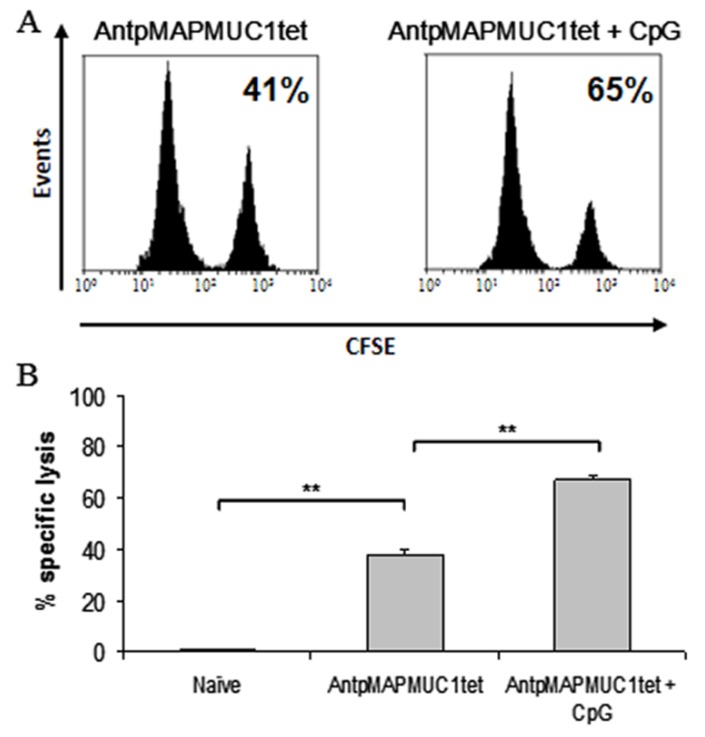
Long term cellular immune responses in AntpMAPMUC1tet immunised mice, measured as in vivo CTL killing assays. (**A**) C57BL/6 mice were immunised i.d. with PBS, 100 µg AntpMAPMUC1tet or 100 µg AntpMAPMUC1tet + 50 µg CpG and the percent MUC1K^b^-specific lysis was determined eight days after immunisation calculated as: {[1 − (ratio CFSE^low^/CFSE^high^ of PBS mice/ratio CFSE^low^/CFSE^high^ of immunised mice)] × 100}. Representative histograms are shown. (**B**) Data is presented as mean % of killing ± SEM (*n* = 6). ** *p* <0 .001.

To detect long termMUC1K^b^ restricted lysis C57BL/6 mice were injected with CFSE labelled cells pulsed with SAPDTRPAP peptide as described previously. Mice pre-immunised with AntpMAPMUC1tet + CpG generated significantly greater (*p* < 0.001) MUC1K^b^-specific lysis than mice immunised with AntpMAPMUC1tet peptide alone, 41 ± 2 vs. 65 ± 2% (Figure 10A,B).

### 2.10. Generation of MUC1-Specific Antibodies

**Figure 11:**
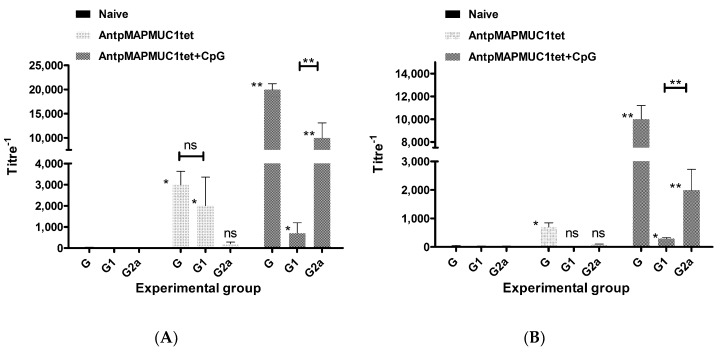
Short and long term MUC1-specific total IgG, IgG1 and IgG2a subclass antibody responses. C57BL/6 mice were injected on day 0, 10 and 17 i.d. with PBS, 100 µg AntpMAPMUC1tet or 100 µg AntpMAPMUC1tet + 50 µg CpG and 14 days after last immunisation mice (short term) (**A**) or 40 days after the last immunisation (long term) (**B**) were bled and total IgG, IgG1 and IgG2a antibody responses to Cp13-32 (MUC1 VNTR) were determined via ELISA. Data presented as mean titre ± SEM (*n* = 4). Statistical difference compared to naïve mice: * *p* < 0.05; ** *p* < 0.001.

At the time of ELISpot assay sera was also collected from C57BL/6 mice to determine antibody responses 2 weeks and 40 days after the last immunisation. The levels of IgG subclasses were analysed, as they provide an indication of the Th1 versus Th2 nature of the immune response. Mice immunised with AntpMAPMUC1tet generated predominantly IgG1 responses significantly greater (*p* < 0.001) than that of PBS injected mice 2 weeks post immunisation (Figure 11A). Mice immunised with AntpMAPMUC1tet + CpG generated significantly greater total IgG antibody titres (*p* < 0.05) than AntpMAPMUC1tet alone and were predominantly of the IgG2a subclass.

To ascertain longer term antibody responses immunised mice were bled 40 days after last immunisation and sera analysed for total IgG, IgG1 and IgG2a isotypes. The levels of anti-MUC1-specific antibody was reduced in the AntpMAPMUC1tet immunised group however, AntpMAPMUC1tet with CpG sustained higher levels of anti-MUC1 antibody and were predominantly of the IgG2a subclass (Figure 11B).

## 3. Discussion

Various therapeutic approaches are being developed and investigated to improve the treatment for cancer patients. A prophylactic or therapeutic vaccine for cancer would be of great benefit. Delivery of target antigen into professional antigen presenting cells (APC) is the first step for inducing antigen-specific T cell responses. Exogenous antigens taken up by endocytosis are transferred to the lysosomal compartment, processed and presented by MHC class II for T cell stimulation. For presentation by MHC class I exogeneous antigens must enter the cytoplasmic compartment for processing by the proteasome for loading class I molecules in the endoplasmic reticulum. Various strategies have been used to delivery antigens into professional antigen presenting cells [3,50,51,52,53,54]. In addition to cytoplasmic delivery to dendritic cells successful priming of T cells is dependent upon two signals one from interaction of peptide loaded MHC class I molecule with the T cell receptor and the other from co-stimulatory molecules CD80 and CD86 binding to CD28 on T cells.

Co-vaccination with adjuvants is widely investigated as a means to further enhance the immune response [55]. TLR are a family of conserved pattern recognition receptors found on several cell types, including DCs, that recognise specific molecular patterns present in pathogens such as bacteria, virus or fungi [56,57]. The activation of TLR by their corresponding ligands on DCs results in the induction of a mature phenotype of DCs, inducing the up-regulation of MHC and co-stimulatory (CD80 and CD86) molecules and the production of inflammatory cytokines (IL-12, type I IFN) for optimal T cell priming, thus potentially augmenting T cell stimulatory capacity [47]. Linear synthetic penetratin or Tat CPP containing peptide or protein immunogens internalise into DC, engender potent immune responses and anti-tumour effects. Mechanistic studies, utilising biochemical inhibitors, have shown that these immunogens are taken up by a combination of direct translocation into the cytoplasm or via an energy dependent endocytic mechanism. The CPP-mediated endocytosed antigens are processed and presented by MHC class II, as well as MHC class I via cross-presentation pathway [4,29,43]. Interestingly, penetratin or Tat-based immunogens do not mature or activate DC or induce cytokine production but capable of inducing therapeutic cytotoxic T cell responses without any adjuvants. However, several studies have shown increased anti-tumour activity when CPP-based immunogens are co-immunised with a TLR agonist [24,42].

Thus far CPP based immunogens have included only linear synthetic peptides, recombinant fusion proteins or covalent conjugates. For this study we designed a novel branched tripartite synthetic peptide incorporating penetratin to facilitate cytoplasmic delivery, a tetanus toxoid universal cell epitope for CD4 help which should enhance long term immune responses and a single repeat from the MUC1 tumour associated antigen which incorporates several Tc epitopes (Figure 1A).

Penetratin or Tat containing immunogens do not mature DC in vitro or in vivo and similarly AntpMAPMUC1tet fails to activate DC (Figure 4). Here we also demonstrate that like other Tat or penetratin-based CPP the tripartite CPP can internalise antigenic peptides into DC (Figure 2C) [29,43,44]. In contrast to the tripartite peptide, alone co-administration of the tripartite peptide with CpG stimulates maturation of DC in vivo, which is evident by elevated expression of the co stimulatory molecules CD40, CD80, CD86 and MHC II (Figure 4). Previously, it has been shown that immunogens incorporating Antp is rapidly uptaken by DC in an ATP dependent manner, and processed by a proteasome and TAP independent pathway for presentation and loading onto MHC class I molecules [29,43]. Here we show that in vivo AntpMAPMUC1tet selectively accumulate in DC in the lymph node following i.d. immunisation (Figure 3). Therefore, given DC proficiency in cross presentation, it is possible that the efficiency by which Antp incorporating peptides are presented by MHC class I may be due, in part, to their high specificity for DC. Furthermore, mice vaccinated with AntpMAPMUC1tet + CpG show enhanced antigen specific IFN-γ and IL-4 T cell responses compared with AntpMAPMUC1tet vaccination alone and induced delayed tumour growth. Vaccination generated long term antibody and promoted a Th1 response, characterised by a higher ratio of IgG2a antibody/IgG1 antibodies (Figure 11B). Previously topical application of a synthetic peptide of penetratin fused to the OVA CTL epitope SIINFEKL, or penetratin chemically conjugated to a 4-armed multiple antigen peptide containing co administered with CpG was shown to confer tumour protection against E.G7-OVA tumour cells [32]. Similarly, mice vaccinated with TAT-CEA + CpG showed enhanced CTL activity and induced a higher ratio of IgG2a/IgG1 CEA specific antibodies compared with CEA alone, CEA + CpG or TAT-CEA immunisation [58] and significantly higher survival rates in a tumour model using MC38/CEA2 [58]. Results presented here with the novel branched tripartite peptide concur with those of Woo et al. (2008) where AntpMAPMUC1tet + CpG immunisation was shown to induce both a potent cellular and humoral immune response. Mice immunised with AntpMAPMUC1tet + CpG generated significantly higher cellular and humoral responses against MUC1 epitopes compared to both AntpMAPMUC1tet and PBS groups in C57BL/6 mice (Figure 5, Figure 6 and Figure 11). Moreover, only mice immunised with AntpMAPMUC1tet + CpG showed delayed tumour growth when challenged with the melanoma B16 MUC1 expressing tumour cell line (Figure 8). Mice immunised with AntpMAPMUC1tet alone did not confer an anti-tumour response.

Although CTLs are recognized as the effector T cells in an anti-tumour response, CD4^+^ Th cells have a significant role in immunity against tumours, necessary for the activation of antigen specific effector cells and recruitment of cells of the innate immune system [59,60]. Both AntpMAPMUC1tet and AntpMAPMUC1tet + CpG immunisation generated significantly higher IL-4 responses against tetanus toxoid when compared to control group.

A Th response can be defined as Th1 or Th2, depending on phenotype and cytokine production [60]. The ability of TLR activated APC to activate CD4^+^ T cells and shape a Th1 immune response has been well described [61]. Mice immunised with AntpMAPMUC1tet + CpG generated significantly greater total IgG antibody titres than AntpMAPMUC1tet. Moreover, AntpMAPMUC1tet + CpG immunisation promoted significant levels of both IgG1 and IgG2a isotypes compared to naïve mice (Figure 11B). Together with the capacity to generate an IL-4 response to tetCD4 as detected by ELIspot assay, it is clear AntpMAPMUC1tet + CpG generates both a Th1 and Th2 response.

The generation of long lasting memory CD8^+^ T cells in vivo is essential for a host to defend against a tumour. Evidence has suggested that the quantity, and prolonged existence of memory CD8^+^ T cells, is determined by the type and strength of signals that T cells received [62]. In this study the ability of co vaccination with CpG to induce long lasting cellular immunity was also compared. Analysis of long term MUC1 specific in vivo lysis revealed that AntpMAPMUC1tet + CpG immunisation promoted greater antigen specific killing than AntpMAPMUC1tet immunisation alone (Figure 10A,B). Moreover, mice immunised withAntpMAPMUC1tet + CpG generated long term IgG and IgG2a responses (Figure 11B). Overall this data suggests that the addition of CpG promoted long lasting memory T cells in vivo. Given AntpMAPMUC1tet alone does not promote augmented costimulatory molecule expression, the enhanced co stimulation provided by DC may in part explain the more robust T cell response and delayed tumour growth conferred when the vaccine is administered with CpG. Such an adjuvant associated property is clearly desirable for boosting immune responses. CpG has been used as a vaccine adjuvant in many clinical trials and considered reasonably safe [63]. However, several trials have noted increased frequency of mild-moderate dose-related local adverse effects such as flu-like symptoms and it use may require close monitoring of patients.

In conclusion Antp can be used to deliver tripartite branched synthetic peptides incorporating multiple CD8 and CD4 T cell epitopes. Moreover, co-immunisation with the adjuvant CpG promotes enhanced antigen specific IFN-γ and IL-4 T cell responses, induces a Th1 response, characterised by a higher ratio of IgG2a /IgG1 antibodies and delays tumour growth in vivo. Furthermore, vaccination generated a long term CD8^+^ memory response. The validation of this form of antigen delivery enables the ready design of other novel tailor made self adjuvanting immunogens, containing Antp and Th epitope, with an appropriate polyepitope peptide of single or multiple TAA specificity for immunotherapy for various cancers.

## 4. Materials and Methods

### 4.1. Peptides

Linear peptides were synthesized by Genescript Corporation (San Francisco, CA, USA). The purity of peptides was determined by mass spectrometry and high performance liquid chromatography and were >95% pure. Antp (penetratin) is a 16 amino acid (RQIKIWFQNRRMKWKK) *Antennapedia* peptide. MUC1K^b^ (SAPDTRPAP) and MUC1A2 (STAPPAHGV) are H-2K^b^ and HLA-A2 CTL epitope peptides, respectively from the MUC1 VNTR region. C-PAHGVTSAPDTRPAPPGSTAP (Cp13-32) is peptide from the MUC1 VNTR repeat containing the MUC1 H-2K^b^ epitope. Tetanus toxoid peptide (tetCD4) (QYIKANSKFIGITEL) is a universal CD4 T helper epitope and RQIKIWFQNRRMKWKKQYIKANSKFIGITEL (Antptet) is a fusion of Antp with tetCD4. The branched tripartite peptide AntpMAPMUC1tet (Figure 1A), with PGSTAPPAHGVTSAPDTRPAPGS, RQIKIWFQNRRMKWKKENK and QYIKANSKFIGITEL(C-terminus) was synthesised by Quality Controlled Biochemicals (QCB, Hopkinton, MA, USA), and was >85% pure.

### 4.2. Conjugation of AntpMAPMUC1tet to Fluorochromes

To prepare Fluorescein-labelled conjugates, AntpMAPMUC1tet was dissolved in 0.2 M sodium bicarbonate and fluorescein isothiocyanate (FITC), (Sigma-Aldrich, St. Louis, MO, USA) dissolved in DMSO was added at a 2.5 fold molar excess. The solution was reacted for 16 h at RT and dialysed overnight (O/N) in PBS at 4 °C to remove unreacted FITC. To prepare Alexa-488-labelled conjugates for confocal microscopy experiments, AntpMAPMUC1tet was dissolved in 0.2 M sodium bicarbonate buffer and labelled with a 2.5 molar excess of the N-hydroxysuccinimide ester of Alexa Fluro 488 dye dissolved in DMSO (Molecular Probes, Eugene, OR, USA). To separate peptide from unreacted Alexa Fluro dye reagent the solution was passed through a PD-10 column.

### 4.3. Mice and Immunisations

C57BL/6 mice, aged 6–10 weeks were purchased from the animal facilities of the Walter and Eliza Hall Institute (Parkville, Australia). HLA-A*0201/K^b^ transgenic mice (HLA-A2) were bred in the facilities of the Burnet Institute (Victoria, Australia). All mice were maintained in the animal house facilities of the Burnet Institute (Victoria, Australia) or RMIT University (Victoria, Australia). HLA-A2 transgenic mice express a chimeric class I molecule that consists of α1 and α2 domains of HLA-A2.1 and the α3 transmembrane and cytoplasmic domains of H-2K^b^ [64].

For immunisation experiments mice were injected three times on days 0, 10 and 17 intradermally (i.d.) at the base of tail. Mice were injected with 100 µg AntpMAPMUC1tet. All animal experiments were approved by the Austin Health and RMIT animal ethics committees (Approval No: E/0842/2009/F).

### 4.4. Adjuvants

CpG-ODN 1668 (5′-TCC ATG ACG TTC CTG ATG CT-3′) with phosphorothioate linkages was synthesized by Geneworks (Adelaide, Australia) and dissolved in sterile PBS and stored at −20 °C.

For immunisation experiments peptides were mixed with CpG (50 µg) and co administered with the peptide into mice three times on days 0, 10 and 17 i.d. at the base of tail.

### 4.5. ELISA

Peptides were coated on a polyvinyl chloride (PVC) microtiter plate at 10 µg/mL in buffer (0.2 M NaHCO_3_ buffer, pH 9.6) overnight at 4 °C and non-specific binding was blocked with 2% bovine serum albumin (BSA) for 1 h at RT. After washing (0.05% Tween 20/phosphate buffered saline (PBS)), serial dilutions of the anti-MUC1 antibody, BC2 recognizing SAPDTRPAP (MUC1K^b^) or 1.3.14 recognizing STAPPAHGV (MUC1A2) were added and incubated for a further 1 h at RT. Plates were then washed and bound antibody was detected using HRP-conjugated sheep anti-mouse antibody (Amersham, UK). Responses were detected with TMB substrate solution (Invitrogen, Carlsbad, CA, USA) and stopped with 1M HCl. Absorbance was read at 450 nm.

### 4.6. Generation of Bone Marrow Derived Dendritic Cells (BMDC)

Bone marrow cells from C57BL/6 female mice were collected by flushing the tibias of hind legs and treated with ACK lysis buffer (0.15 M NH_4_Cl, 1 mM KHCO_3_ and 0.1 mM Na_2_EDTA) to lyse erythrocytes. Cells were washed and cultured at 5 × 10^5^ cells/ml in 24 well plates with complete RPMI-1640 medium (10% (*v*/*v*) heat inactivated fetal calf serum, 4 mM l-glutamine, 100 U/mL penicillin, 100 µg/mL streptomycin sulphate and 100 µM β-mercaptoethanol) with 10 ng/mL of recombinant mouse granulocyte macrophage colony-stimulating factor (GM-CSF) and IL-4 (BD Pharmingen, San Diego, CA, USA). At day 6 cells were >80% CD11c^+^.

### 4.7. Internalisation into BMDC

Day 6 cultured C57BL/6 BMDC were pulsed at 5, 20, 100 and 200 µg/mL with peptides for a 1 h at 37 °C in serum free media. Due to cell fixation causing artefactual uptake of CPP peptides all uptake experiments were performed by measuring the surface and intracellular presence, with results expressed as intracellular percentage—surface percentage [23,44]. For surface staining (detect extracellular peptide) DC were washed with 0.5% BSA/PBS and incubated with the anti-MUC1 monoclonal antibody BC2 (diluted in 0.5% BSA/PBS) for 30 min at 4 °C. Cells were washed and anti-(Fab’)_2_-FITC (Chemicon, Melbourne, Australia) was added in 0.5% BSA /PBS for a further 30 min at 4 °C. For intracellular staining (detect extracellular and intracellular peptide) cells were fixed with 2% paraformaldehyde for 10 min at room temperature and washed and permeabilised with 0.25% saponin/PBS for 10 min. Cells were then stained as above in 0.25% saponin/PBS. Kinetics of uptake was also assessed by adding peptides for various times (5–360 min) at a fixed concentration at 37 °C. DC were resuspended in PBS and analysed by flow cytometry (BDCanto, BD Biosciences, San Jose, CA, USA). Results are expressed as intracellular percentage—surface percentage. Isotype controls were used as negative control (background) staining.

### 4.8. In Vitro Maturation

Stimulants were added to day 4 GM-CSF grown BMDC culture. After 18 h incubation cells were harvested washed and stained with anti-CD40, anti-CD80 and anti-CD86 conjugated to FITC, together with the DC marker anti-CD11c (BD Pharmingen) for 20 min at 4 °C in 2% FCS/PBS. Cells were washed and analysed via flow cytometry. Live BMDC population was gated based on the PI negative cells and CD11c^+^ [65].

### 4.9. In Vivo Maturation

C57BL/6 mice were injected i.d. in the footpad with peptides. LPS (1 µg/mL) and PBS were used as positive and negative controls respectively. 18 h later popliteal lymph nodes were isolated and pooled (*n* = 4) and stained with CD11c-APC and maturation markers (CD40, CD80, CD86, and MHC class II) assessed by flow cytometry. Live cells were gated on PI staining and cells gated on CD11c^+hi^ staining [48].

### 4.10. In Vivo Binding Specificity

C57BL/6 mice were injected i.d. with 100 µg FITC-labelled AntpMAPMUC1tetFITC or PBS in footpads for 18 h. Popliteal lymph nodes cells were isolated and pooled from mice (*n* = 3) and cells labelled with fluorochrome-conjugated anti-CD3, anti-CD19, anti-CD11c and anti-F4/80 (BD Pharmingen) to separate T cells, B cells, CD11c^hi^ DC and F4/80^hi^ macrophages respectively [45,48].

To assess binding of DC subsets popliteal lymph nodes were pooled (*n* = 4) and resuspended in free balanced salt solution (BSS) with 0.1 M EDTA (pH 7.2) to dissociate T cells from DCs and incubated with CD11c microbeads (10 µL beads/10^7^ cells) (Miltenyi Biotec, Bergisch Gladbach, Germany) for 30 min at 4 °C. Cells were washed and resuspended in BSS and passed through AutoMACS columns (Miltenyi Biotech, Germany). Purity of cells (CD11c^+^) was >90%.

To differentiate DC subpopulations, cells were stained with biotin-labelled anti-DEC205 and streptavidinPE-Cy7 and anti-CD8-PE together with anti-CD11cAPC and analysed for FITC expression. Three major subsets in the popliteal lymph nodes subsets were identified: CD8^–^ DEC205^–^ (immature), CD8^+^ DEC205^–^ (immature) and CD8^low^ DEC205^+^ DCs (migratory) [46].

### 4.11. Enzyme-Linked Immunosorbent Spot-Forming Cell Assay (ELISpot)

To determine effector immune response splenocytes from immunised C57BL/6 and HLA-A2 transgenic mice immunised i.d. on days 0, 10 and 17 were isolated 14 days after final immunisation and assessed by ELISpot for IFN-γ and IL-4 secretion. To determine long term immune responses splenocytes from C57BL/6 mice were isolated 40 days after final immunisation. MultiScreen filter plates (Millipore, Billerica, MA) were coated with 70 µL 5 µg/mL anti-mouse IFN-γ antibody (AN18) or anti-mouse IL-4 antibody (Mabtech, Stockholm, Sweden) overnight at 4 °C. Plates were washed six times with sterile PBS and blocked with 200 µL complete RPMI media for 2 h at 37 °C. Spleen cells (5 × 10^5^/well) in 100 µl of complete medium were incubated with 20 µg/mL various recall antigens for 18 h in IFN-γ ELISpot and 24 h in IL-4 ELISpot. Recall antigens included SAPDTRPAP, STAPPHAGV and tetCD4. Concanavalin A (ConA) (1 µg/mL) or cells alone were used as positive and negative controls respectively. Triplicate wells were set up for each condition. Cells were discarded after washing (PBS) and 1 µg/mL biotinylated anti-mouse IFN-γ antibody or biotinylated anti-IL-4 antibody (Mabtech) was added for 2 h at RT. The plates were washed with PBS and 1ug/mL streptavidin-alkaline phosphatase (AP) (Mabtech) added at room temperature for 2 h. Spots of activity detected using a colorimetric AP-conjugate substrate kit (Bio-Rad Laboratories, Foster City, CA, USA). Cytokine spots were counted with an AID ELISpot Reader system (Autoimmun Diagnostika GmbH, Strassberg, Germany). Data are presented as mean spot-forming units (SFU) per 5 × 10^5^ cells ± standard error of the mean (SEM).

### 4.12. Antibody ELISA

Sera was collected from C57BL/6 and HLA-A2 transgenic mice 14 or 40 days after final immunisation via orbital bleed. Red blood cells pelleted by centrifugation at 13,000 rpm for 10 min and serum was aspirated and stored at −20 °C until use.

The MUC1 VNTR peptide Cp13-32 (C-PAHGVTSAPDTRPAPGSTAP) was coated onto PVC microtiter plates at 10 µg/mL in 0.2M NaHCO_3_ buffer, pH 9.6, overnight at 4 °C. After washing (0.05%Tween 20/PBS), non-specific binding was blocked with 2% BSA/PBS for 1 h at RT. Serial dilutions of sera were added (in 2% BSA/PBS) and incubated for a further 2 h at RT. Plates were washed and bound antibody detected using horseradish peroxidase-conjugated sheep anti-mouse IgG (Amersham, UK). To determine Ig subclasses plates were washed and bound antibody detected using biotin conjugated sheep anti-mouse IgG1 or IgG2a (BD Pharmingen). Plates were washed as described above and streptavidin HRP added for 1 h at RT. Responses were detected with TMB substrate solution and stopped with 1M HCl. Absorbance was read at 450 nm. End-titre was defined as the last value in the titration to remain above the corresponding control value, where the control was calculated as the mean OD values + 2 SD of naive mouse serum samples (4 mice) at each titration point.

### 4.13. Confocal Microscopy

Day 5 BM-CSF and IL-4 grown BMDC (5 × 10^4^ cells) were seeded in eight well chambered cover glasses (Nunc) O/N at 37 °C. The next day culture media was aspirated and replaced with 200 µL Hank’s Buffered Salt Solution (HBSS) and pulsed with Alexa-labelled AntpMAPMUC1tet peptide for 2 h 37 °C. Hoechst nuclear stain (10 µg/mL) (Molecular Probes) was added in for 15 min at 37 °C. Cells were washed extensively and visualized using laser scanning microscope [43]. To prevent fixation artefacts all measurements of peptide uptake were performed with living, non-fixed cells. For inhibition of internalisation BMDC were pre incubated with and without cytochalasin D (Sigma, Gillingham, UK) at 10 µg/mL which blocks phagocytosis for 45 min at 37 °C prior to pulsing with AntpMUC1tet.

### 4.14. Generation of Human Monocyte Derived Dendritic Cells (MoDC)

In order to generate MoDC, peripheral blood mononuclear cells (PBMCs) were isolated from the buffy coat of healthy individuals (Australian Red Cross) [66]. Use of de-identified buffy coats from the Australian Red Cross was approved by the Alfred Health Research Ethics Committee (Approval No: 162/09). Buffy coats were diluted 1:1 in sterile PBS and PBMC isolated by Ficoll-Paque density gradient centrifugation (GE Healthcare Bio-sciences, Uppsala, Sweden). Isolated PBMCs were resuspended at 10^7^ cells/20 uL of anti-CD14 microbeads (Miltenyi, Germany) in EDTA-PBS/0.5%BSA buffer and incubated at 4 °C for 20 min. Cells were washed and passed through the Automacs cell-sorting system and CD14^+^ monocytes collected by positive selection (AutoMACS). CD14^+^ cells cultured with 20 ng/mL recombinant human GM-CSF and 10 ng/mL IL-4 for 6 days (BD Pharmingen). Purity of MoDC was confirmed by staining with anti HLA-DRPerCP and Lin^–ve^FITC cocktail (CD3, CD14, CD16, CD19, CD20, CD56) (BD Pharmingen).

### 4.15. Generation of Tetanus Toxoid-Specific T Cell Lines

PBMC were separated from buffy coats via density gradient centrifugation using Ficoll-Paque PLUS (GE Healthcare). PBMC were resuspended at 5 × 10^6^/mL in complete AB media (RPMI1640, 10% AB serum, Pen/Strep, HEPES, L-GLUT, NEAA, Sodium Pyruvate (Invitrogen), 2-mercaptoethanol) was stimulated with 20 µg/mL of tetCD4 and 5 µg/mL CpG in a 24 well plate. Three days later, another 1ml complete AB media supplemented with 25U/mL IL-2 (R & D Systems), 10 ng/mL IL-15 and 10 ng/mL IL-7 (Peprotech) was added. Seven-10 days after initial priming, cells were re-stimulated with autologous MoDC pulsed with 20 µg/mL tetCD4 peptide. On the following day, 1ml supernatant was exchanged for fresh complete AB media containing 25 U/mL IL-2. This was repeated every 3–4 days. tetCD4-specific T cell lines were generated by FACS sorting. IFNγ secreting cells specific for tetCD4 peptides were sorted using the FACS Aria following a 4 h incubation with tetCD4-pulsed MoDC. IFNγ secreting cells were identified using the IFNγ secretion and detection assay (Miltenyi Biotech) performed according to manufacturer’s protocol.

### 4.16. Analysis of Antigen-Specific T Cell Responses

MoDC (2 × 10^5^/well) were prepared by pulsing with an irrelevant peptide or tetCD4 (20 µg/mL) for 1hr in serum free media. Pulsed MoDC were added at about a 1:5 ratio (4 × 10^4^/well) in 100 µL to the duplicate T cell wells and incubate for 1hr at 37 °C 5% CO_2_. 50 µL media containing Golgi-Stop (0.1 µL per 200 uL T cell/MoDC co-culture) was added and cells were stained for surface markers CD8 and CD4, fixed and permeabilised, then stained for accumulation of intracellular IFNγ using CD4 APC-Cy7, CD8 FITC and IFNγ PE-Cy7(BD) with the Cytofix/CytoPerm Kit (BD). Antigen-specific T cells were identified by flow cytometry comparing IFNγ^+^ CD4 and CD8 T cells in the presence of the peptide of interest with the irrelevant peptide controls.

### 4.17. Prophylactic Tumour Protection

Groups of 8 C57BL/6 mice were injected intradermally (i.d.) at the base of tail with PBS, 100 µg of AntpMAPMUC1tet or 100 µg AntpMAPMUC1tet with 50 µg CpG on days 0, 10 and 17. Seven days following the last immunisation, mice were shaved on the abdominal area and challenged subcutaneously (s.c.) with 2 × 10^5^ MUC1-B16 melanoma cells suspended in 100 µL PBS. Expression of MUC1 on MUC1-B16 cells was confirmed by flow cytometry prior to challenge. The growth of tumours was monitored by measuring the two perpendicular diameters using callipers and the results were expressed as their product.

### 4.18. Statistical Analysis

Mean values were compared using unpaired t-tests and one-way ANOVA using Graphpad InStat version 3.0a (GraphPad Software, San Diego, CA, USA). Two *p*-value thresholds were used for immunogenicity assays: * *p* < 0.05 to indicate a significant difference, ** *p* < 0.001 to indicate a highly significant difference.
